# Lactate and base excess (BE) as markers of hypoperfusion and mortality in traumatic hemorrhagic shock in patients undergoing Damage Control: a historical cohort

**DOI:** 10.1590/0100-6991e-20243699-en

**Published:** 2024-06-14

**Authors:** FERNANDA BAEUMLE REESE, FLAVIA CASTANHO HUBERT, MARIANA BRUINJE COSENTINO, MIRELLA CRISTINE DE OLIVEIRA, ÁLVARO RÉA, RAFAELLA STRADIOTTO BERNARDELLI, JORGE EDUARDO MATIAS

**Affiliations:** 1 - Universidade Federal do Paraná, Programa de Mestrado - Curitiba - PR - Brasil; 2 - Universidade Federal do Paraná, Complexo Hospital de Clínicas da Universidade Federal do Paraná - Curitiba - PR - Brasil; 3 - Hospital do Trabalhador, Hospital do Trabalhador - Curitiba - PR - Brasil; 4 - Pontifícia Universidade Católica do Paraná (PUC-PR), Centro de estudos e pesquisa em Terapia Intensiva (CEPETI) - Curitiba - PR - Brasil; 5 - Universidade Federal do Paraná, Departamento de Clínica Médica - Curitiba - PR - Brasil

**Keywords:** Shock, Hemorrhagic, Acidosis, Lactic, Multiple Trauma, Blood Gas Analysis, Choque Hemorrágico, Traumatismo Múltiplo, Gasometria Arterial, Acidose Lática

## Abstract

**Introduction:**

hemorrhagic shock is a significant cause of trauma-related deaths in Brazil and worldwide. This study aims to compare BE and lactate values at ICU admission and twenty-four hours after in identifying tissue hypoperfusion and mortality.

**Methods:**

examines a historical cohort of trauma patients over eitheen years old submittet to damage control resuscitation approch upon hospital admission and were then admitted to the ICU. We collected and analyzed ISS, mechanism and type of trauma, need for renal replacement therapy, massive transfusion. BE, lactate, pH, bicarbonate at ICU admission and twenty-four hours later, and mortality data. The patients were grouped based on their BE values (≥-6 and <-6mmol/L), which were previously identified in the literature as predictors of severity. They were subsequently redivided using the most accurate values found in this sample. In addition to performing multivariate binary logistic regression. The data were compared using several statistical tests due to diversity and according to the indication for each variable.

**Results:**

there were significant changes in perfusion upon admission to the Intensive Care Unit. BE is a statistically significant value for predicting mortality, as determined by using values from previous literature and from this study.

**Conclusion:**

the results demonstrate the importance of monitoring BE levels in the prediction of ICU mortality. BE proves to be a valuable bedside marker with quick results and wide availability.

## RESUMO

**Introdução:**

o choque hemorrágico é a principal causa reversível de morte no trauma no Brasil e no mundo.

**Objetivo:**

comparar o valor de *BE* ao do lactato na admissão da UTI e vinte e quatro horas após o internamento na identificação de hipoperfusão tecidual e predição de mortalidade

**Método:**

coorte histórica de pacientes traumatizados, maiores de dezoito anos, submetidos à estratégia de controle de danos na admissão hospitalar, seguido de internamento em UTI. Foram coletados e analisados *ISS*, mecanismo e tipo de trauma, necessidade de terapia de substituição renal e transfusão maciça; *BE*, lactato, pH e bicarbonato coletados na admissão da UTI e vinte e quatro horas após, e a mortalidade. Os pacientes foram divididos em grupos conforme valores de *BE* (≥-6 e <-6mmol/L) já descritos na literatura como preditores de gravidade, e após redivididos de acordo com os valores de melhor acurácia encontrados nesta amostra, além de realização de regressão logística binária multivariada. Os dados foram comparados através de diversos testes estatísticos devido a diversidade e conforme a indicação para cada variável.

**Resultados:**

houve alterações perfusionais impactantes já na admissão da UTI. *BE* manteve-se com valor estatisticamente significativo para predição de mortalidade tanto quando utilizado os valores já conhecidos da literatura como quando aplicados os valores neste estudo identificados.

**Conclusão:**

valores de *BE* e de lactato foram capazes de predizer hipoperfusão tecidual e mortalidade nos dois momentos estudados, quando comparados, o *BE* tem boa performance como preditor de mortalidade, com rápido resultado e ampla disponibilidade.

## INTRODUCTION

Hemorrhagic shock secondary to trauma is the leading cause of reversible death in Brazil and worldwide, and the abdomen is an important source of bleeding^1^ ^,^ ^2^. Volume loss leads to a decrease in blood flow in the microcirculation, generating hypoperfusion and consequent anaerobic metabolism due to cellular hypoxemia, causing metabolic acidosis^3^ ^,^ ^4^. Reaching this diagnosis early is essential, but it remains a challenge, especially in young patients who have adequate physiological reserve and do not present early changes in clinical signs^5^. 

The initial phase of this type of shock may be silent and even go unnoticed in the initial clinical evaluation. However, there may already be severe metabolic alterations and occult hypoperfusion, which makes this diagnosis mandatory, as well as early action in the face of such events^3^ ^-^ ^6^. Therefore, estimating the patient’s volume loss to adjust replacement remains a major challenge at the bedside^5^.

The care and therapeutic management of a patient with severe abdominal trauma is extremely difficult, hance the need for collaborative work between the clinical and surgical teams^7^. Due to the complexity of the lesions found, physiological derangement, and the severity of the patient, damage control surgery is necessary, controlling bleeding and contamination of the cavity, with temporary closure for a later definitive surgical approach, which is performed after the patient’s clinical and laboratory stabilization^8^. Those who require this surgical strategy in general suffered considerable blood loss and consequent tissue hypoperfusion and metabolic acidosis, in addition to hypothermia, coagulopathy, and hypocalcemia^8^ ^,^ ^9^.

Expedite hemodynamic management is essential, with the use of blood components, whole blood, crystalloids, and eventually synthetic blood products for clinical and hemodynamic stabilization^3^ ^,^ ^10^ ^-^ ^12^. The use of vasoactive drugs is concomitant, if necessary, to maintain an adequate mean arterial pressure, ensure blood flow, and avoid worsening of tissue hypoperfusion^13^. 

A pioneering clinical study correlated the base excess (BE) value of hospital admission with the volume loss of traumatic hemorrhagic shock and with the amount of volume to be administered^16^. This fact is relevant, since BE is a complementary test that is quickly accessible and available in emergency and intensive care settings. 

The objective of this study is to compare the value of BE to that of lactate at ICU admission and twenty four hours after hospitalization, to identify patients with tissue hypoperfusion who require immediate clinical and hemodynamic management, in addition to analyzing its value as a predictor of mortality at both moments. 

## METHODS

This is a historical cohort of trauma patients who underwent abdominal damage control strategy, treated in the emergency room of the Worker’s Hospital, a trauma referral hospital in the Brazilian state of Paraná, between January 2012 and December 2018. The study was approved by the Ethics in Research Committee of the Worker’s Hospital (CAAE: 89852718.7.0000.5225 and Opinion Number: 2.751.732). We included all patients over eighteen years of age who were admitted to the ICU after the surgical procedure.

We collected data on age, sex, mechanism and type of trauma, comorbidities, trauma score (Injury Severity Score - ISS), need for renal replacement therapy and massive transfusion, as well as pH, bicarbonate, BE and lactate values at ICU admission and twenty four hours after, and ICU mortality.

To analyze the impact of hypoperfusion estimated by the BE on the various parameters studied, we divided patients, as brought by previous work^17^, into two categories, according to the result of the BE (≥-6 and <-6mmol/L), and at two distinct and defined moments (ICU admission and 24 hours later). At those same moments, we also divided patients according to BE values reaching better sensitivity and specificity, as identified in the present study. 

We described categorical variables (sex, mechanism of trauma, type of trauma, need for renal replacement therapy, and massive transfusion) by frequency and percentage. The quantitative variables that presented normal distribution according to the Kolmogorov-Smirnov test are described as mean and standard deviation, while those that did not display normality are described as median and interquartile range. 

We compared normally distribution quantitative variables between groups by different cutoffs both in the BE at admission and in the BE at twenty four hours with the Student’s t-test for independent samples, while the quantitative variables not normally distributed were compared between groups with the non-parametric Mann-Whitney test. We used the Fisher’s exact test to assess the need for renal replacement therapy and to compare mortality among patients grouped by BE cutoffs. We applied the Spearman’s correlation test to evaluate the correlation between the BE value at admission and the ISS score.

We analyzed the predictive value of the BE at admission and twenty-four hours after ICU admission, as well as of lactate at the same moments, using a receiver operator characteristic curve (ROC) based on the graphic distribution of the values obtained for sensitivity and specificity, as results described by area under the curve (AUC) and their 95% confidence intervals. By multiplying each sensitivity and specificity value generated by the ROC curve, we identified the point that best discriminates mortality for each of the four variables evaluated.

We fitted four multivariate binary logistic regression models to assess the influence of each of the variables (pH, BE, bicarbonate, and lactate) on mortality, when adjusted for age, sex, and ISS. The variables were not included in a single multivariate model, given the multicollinearity identified between them. The results were expressed as Odds Ratios, 95% confidence intervals, and Wald test significance level.

The level of statistical significance was set at 5% and the data were analyzed using the statistical software IBM SPSS, version 28.0 (SPSS Inc., Chicago, IL, USA). Missing data were not imputed.

## RESULTS

**Figure 1:**
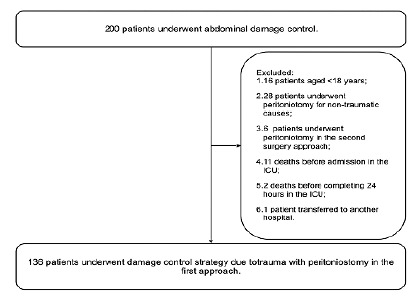
Sampling Flowchart.

During the study period, 200 patients underwent abdominal damage control surgery. After excluding 64 patients due to the established criteria (Figure 1), 136 patients were selected for the study. One patient was excluded due to lack of data regarding the period 24 hours after ICU admission in the medical records.

The mean age of the population was 32.5±11.9 years, with only five individuals over sixty years, and a predominance of males (89%). Of the total sample, 68.4% were victims of open abdominal trauma, gunshot wound (GSW) being the most common mechanism of injury. Among the patients with associated chest trauma, the therapy used was closed chest drainage, and only one patient required thoracotomy to control bleeding.

Most patients with associated orthopedic trauma had fractures of the upper limbs and some of the femur bones. Regarding comorbidities, since they were predominantly young, only one patient with previous non-dialysis chronic renal failure was observed, a post-transplant patient due to hypertensive nephropathy; 24.3% of the patients required hemodialysis. There were also alcoholism and drug addiction (N=5), smoking, systemic arterial hypertension (N=4) and type 2 diabetes (N=3). 

Table 2 shows metabolic and perfusion variables secondary to hemorrhagic shock in patients included in this cohort when they were admitted to the ICU after the surgical procedure and initial hemodynamic management, with very altered BE and lactate medians at the two studied moments, and a very significant clearance of both variables in this period. A state of metabolic acidemia and acidosis is notorious, with altered pH, BE and bicarbonate, especially at admission, but considerable changes ensue after twenty four hours of clinical management.

[Table t1]

**Table 1 t1:** Demographic data of the population studied.

Sample characteristics	n=136
Male sex, n(%)	121 (89,0)
Age, mean ± SD	32,5 ± 11,9
Mechanism of trauma, n(%)	
GSW	74 (54,4)
SW	18 (13,2)
Traffic accident	35 (25,7)
FSL and FOL	9 (6,6)
Type of trauma, n(%)	
Open	93 (68,4)
Closed	43 (31,6)
Associated trauma, n(%)	
TBI	6 (4,4)
Chest trauma	23 (16,9)
Orthopedic trauma	57 (41,9)
ISS, med (IQR)	31 (25 | 38)

GSW: gunshot wound; SW: stab wound; FSL: fall from the same level; FOL: fall from other level; ISS: Injury Severity Score; TBI: traumatic brain injury; SD: standard deviation; Med: median; IQR: interquartile range.

**Table 2:** Laboratory tests at admission to the ICU and twenty-four hours later.

Laboratory Tests	Value at ICU admission (n=136) Med. (IQR)	Value after 24h (n=135) Med. (IQR)
pH	7,240 (7,170 | 7,300)	7,360 (7,290 | 7,420)
Bicarbonate	17,1 (14,8 | 19,1)	18,4 (15,8 | 20,35)
BE	-9,4 (-12,95 | -7,1)	-6,3 (-8,9 | -3,35)
Lactate	4,0 (2,7 | 6,1)	2,7 (1,9 | 4,2) a
Massive transfusion, n (%)	33 (24,3) 33,88 (1,45 | 62,94) 32,13 (11,03 | 51,72)	
BE clearence* in 24 hours % (min | max)		
Lactate clearance* in 24 hours % (min | max)		

SD: standard deviation; med: median; IQR: interquartile range; min: minimum value; max: Maximum value. a n=134. * Clearance = ((value of ICU admission - value after 24 hours)/value of ICU admission) x100.

Table 3 describes the perfusion variables (pH, bicarbonate, and lactate), severity of trauma at admission (ISS), need for renal replacement therapy, and mortality in the BE subgroups (≥-6 and <-6mmol/L), both at admission and at twenty four hours. It is possible to note the clinical relevance of the subdivision of the values of the analyzed parameters according to the degree of alteration in the BE, showing statistical significance for all parameters of tissue perfusion, initial ISS, and mortality at the time of admission to the ICU, which remains significant 24 hours later. 

**Table 3:** Comparison of pH, bicarbonate, and arterial lactate between patients with BE ≥-6 and <-6, both at admission and twenty four hours later, and severity of trauma at admission - ISS.

Variable	Admission BE ≥-6 (n=22)	Admission BE <-6 (n=114)	p-value
ISS, med (IQR)	25 (17.5 | 32.5)	34 (25 | 38)	0,024#
pH, mean ± SD	7,343 ± 0,051	7,209 ± 0,099	<0.001*
Bicarbonate, mean ± SD	21 ± 1.7	15.9 ± 2.9	<0.001*
Arterial lactate, mean ± SD	3 ± 1.6	5.2 ± 3	<0.001*
Hemodialysis, n(%)	2 (9,1)	28 (24,6)	0.16§
ICU mortality, n(%)	3 (13,6)	42 (36,8)	0.046^§^
	BE ≥-6 24h (n=63)	BE <-6 24h (n=72)	p-value
pH, mean ± SD	7,411 ±,073	7,273 ± 0,132	<0.001*
Bicarbonate, mean ± SD	20.7 ± 2.4	15.3 ± 3.8	<0.001*
Arterial lactate, mean ± SD	2.3 ± 1.5	5.1 ± 4.7	<0.001*
Hemodialysis, n(%)	9 (14,1)	21 (29,2)	0.04^§^
ICU mortality, n(%)	15 (23,4)	30 (41,7)	0,029^§^

ISS: Injury Severity Score, SD: standard deviation; med: median; IQR: interquartile range; n: frequency; %: percentage. * Significance of the Student’s t-test, p<0.05. # Significance of the nonparametric Mann-Whitney test, p<0.05. § Fisher’s exact test, p<0.05.

When analyzing the need for renal replacement therapy, hemodialysis, taking into account the BE after twenty four hours, unlike the analysis that occurred with the BE at ICU admission, there was statistical significance, and 29.2% of the patients who remained in the BE group <-6mmol/L after volume optimization required hemodialysis, versus 14.1% in the BE ≥-6mmol/dL group (p=0.04), as shown in Table 3.

**Figure 2:**
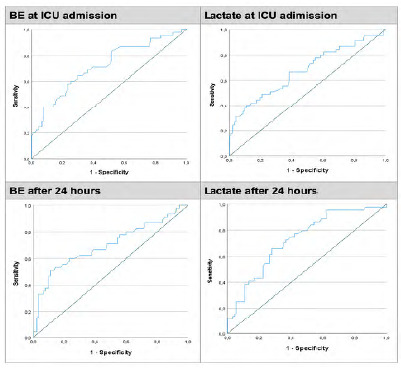
Comparison of BE and lactate as predictors of mortality at ICU admission and after 24 hours.

Figure 2 shows the areas below the curve comparing BE and lactate in the two different moments of analysis of the present study. Both BE and lactate, at ICU admission and twenty four hours later, are good predictors of in-hospital mortality, since the four graphs present areas below the ROC curve greater than 0.5, with good fit. The points with the highest concomitant sensitivity and specificity, i.e., with the highest accuracy, are the BE values at admission -10.35mmol/L (sensitivity 63% and specificity 70% - Figure 2A); lactate at admission 4.43mmol/L (sensitivity 66.7% and specificity 60% - Figure 2B); twenty four hour BE -8.65mmol/L (sensitivity 54.3% and specificity 86.5% - Figure 2C), and twenty four hour lactate 3.05mmol/L (sensitivity 66.7% and specificity 73.0% - Figure 2D).

In the population studied, with severe polytraumatized patients, the best BE cutoff values are higher than those described and recommended in the literature^17^. Therefore, the variables were reclassified according to the BE cutoffs calculated in this series at the two moments described, which are shown in Table 4. It should be noted that none of the previous comparisons lost statistical significance with the cutoffs’ modification, and the BE was a good predictor of the need for renal replacement therapy at ICU admission (Table 4).

**Table 4:** Comparison of pH, bicarbonate, and arterial lactate between patients with BE values according to the ROC curve, at admission and 24 hours later, and severity of trauma - ISS.

Variable	Admission BE >-10.35 (n=80)	Admission BE ≤-10.35 (n=56)	p-value
ISS, med (IQR)	25 (20,25 | 34)	34 (25 | 41)	0,001^#^
pH, mean ± SD	7,294 ± 0,062	7,141 ± 0,088	<0.001*
Bicarbonate, med (IQR)	18,7 (17,4 | 20,0)	14,4 (12,1 | 15,8)	<0.001^#^
Arterial lactate, mean ± SD	3,5 ± 1,6	6,7 ± 3,3	<0.001*
Hemodialysis, n(%)	12 (15)	18 (32,1)	0.022^§^
ICU mortality, n(%)	16 (20,0)	29 (51,8)	<0.001^§^
	24-hour BE >-8.65 (n=98) mean ± SD; Med. (min | max)	24-hour BE ≤-8.65 (n=37) mean ± SD; Med. (min | max)	p-value
pH, med (IQR)	7,38 (7,34 | 7,43)	7,26 (7,18 | 7,33)	<0,001^#^
Bicarbonate, med (IQR)	19,4 (17,9 | 21,4)	13,6 (11 | 15,2)	<0,001^#^
Arterial lactate, med (IQR)	2,2 (1,7 | 3,6)	4,2 (2,8 | 8,7)	<0,001^#^
Hemodialysis, n(%)	13 (13,3)	17 (45,9)	<0,001^§^
ICU mortality, n(%)	21 (21,4)	24 (64,9)	<0,001^§^

Abbreviations: ISS: Injury Severity Score, SD: standard deviation; med: median; min: minimum value; max: maximum value; n: frequency; %: percentage.*Significance of the Student’s t-test, p<0.05. #Significance of the nonparametric Mann-Whitney test, p<0.05.§Fisher’s exact test, p<0.05.

There was also a weak and inverse, though significant, correlation between the BE at admission and the ISS score (RHO coefficient -0.245, p=0.004).

Regarding ICU mortality, we observed that lower pH, BE, and bicarbonate values at ICU admission increase the chance of death, even when each of them is adjusted for age, sex, and ISS (Table 5).

**Table 5:** Multivariate binary logistic regression models of pH, BE, bicarbonate, and lactate in predicting mortality, when adjusted for age, sex, and ISS.

Templates	Model Variables	OR (95% CI) for ICU mortality*	p# value
Model 1 - with pH	pH value at admission	0,003 (0,00005 - 0,14)	0,003
	Male sex (ref. Female)	1,238 (0,316 - 4,853)	0,760
	Age, in years	1,053 (1,019 - 1,09)	0,002
	ISS Score	1,034 (0,997 - 1,074)	0,075
Model 2 - with BE	BE at admission	0,818 (0,737 - 0,908)	0,000
	Male sex (ref. Female)	1,726 (0,416 - 7,16)	0,452
	Age, in years	1,05 (1,014 - 1,088)	0,006
	ISS Score	1,031 (0,992 - 1,071)	0,119
Model 3 - with bicarbonate	Bicarbonate at admission	0,747 (0,647 - 0,862)	0,000
	Male sex (ref. Female)	2,268 (0,528 - 9,751)	0,271
	Age, in years	1,048 (1,011 - 1,085)	0,010
	ISS Score	1,032 (0,992 - 1,073)	0,117
Model 4 - with lactate	Lactate at admission	1,297 (1,113 - 1,511)	0,001
	Male sex (ref. Female)	1,348 (0,335 - 5,421)	0,674
	Age, in years	1,05 (1,014 - 1,088)	0,006
	ISS Score	1,029 (0,99 - 1,068)	0,144

*Odds ratio and 95% confidence interval of the multivariate binary logistic regression model. #Significance of the Wald test.

## DISCUSSÃO

Serum lactate is the most widely used marker of tissue hypoperfusion in scientific studies and also the most studied^18^ and is therefore the natural choice for comparisons with BE, which is quickly obtained, as a marker for the same state of perfusion alteration.

In a recent study, the BE value measured at hospital admission of trauma patients proved to be more accurate than lactate in identifying early hemorrhagic shock, need for volume resuscitation, blood use, and clinical outcome, risk of death^19^. 

Both BE and serum lactate reliably indicate states of tissue hypoperfusion^20^. BE is rapidly altered and maintains a clinical-laboratory correlation similar to that of lactate and is measured quickly and safely in arterial blood gas analysis^21^, which can be performed promptly to diagnose hypoperfusion.

The study population consisted of critically ill patients with complex abdominal lesions, with high ISS (median 31). The literature classifies the severity of trauma according to the ISS as mild with values between 1 and 15, moderate between 16 and 24, and severe when greater than or equal to 25, with a value of 16 considered a critical cutoff^22^ ^,^ ^23^.

BE correlated with patients’ severity at admission as estimated by the ISS, a fact of extreme importance in daily clinical practice, given the easy access to this variable when compared to the calculation of the ISS, a score that requires diagnosis of all anatomical lesions to obtain the result, which cannot be performed quickly at the patient’s admission, and only after the definitive surgical procedure and identification of all the patient’s lesions (Table 3).

Unlike other series in the literature involving abdominal trauma, in this study we included only severe polytrauma patients who underwent damage control strategy, obtaining a total of 136 patients, a number considered significant in the pertinent literature, damage control surgery alone being a risk factor for death in patients with blunt abdominal trauma^7^ ^,^ ^8^ ^,^ ^24^. We also emphasize that 100% of the sample required sedation, invasive mechanical ventilation, vasoactive drugs, and invasive devices when admitted to the ICU. It is estimated that around 10% of severe traumas require damage control surgery in their initial care^25^.

Other data that corroborate the complexity and severity of the population studied in this study are the median elevated BE and lactate levels at ICU admission (-9.4mmol/L and 4mmol/L, respectively). Authors propose a cutoff of -6mmol/L at ICU admission for the BE to discriminate severity, need for blood transfusion, and clinical outcome^15^ ^,^ ^20^ ^,^ ^26^.

BE was introduced in the standard blood gas evaluation in 1960, after about 40 years of research, being a reliable marker of acidosis or alkalosis, regardless of respiratory disorders or compensatory respiratory alterations to keep the patient’s pH in the normal range. It is the amount of acid or base that must be added to the blood to reach a pH of 7.40 under normal conditions (PaCO_2_ 40mmHg). It was also introduced because it was able to identify the severity of the disorder according to changes in its value^27^. 

Some limitations should be pointed out. BE may be within the normal range in some clinical situations, such as exogenous administration of sodium bicarbonate due to alteration of osmolarity and negative ionic compensation of sodium, even in the presence of hyperlactatemia, and in cases of hyponatremia, hypochloremia, and ketosis, due to the same ionic compensation. Clinically, it can be translated with the example: patients with uncontrollable vomiting, children with severe pylorus stenosis. Therefore, the evaluation in conjunction with the patient’s clinical condition and other perfusion variables in this scenario are essential^27^.

The products of anaerobic metabolism, the setting of tissue hypoxia, include lactic acid and other organic acids, which accumulate to generate metabolic acidosis, or a relative “excess acid” in the blood. This “excess acid” is also called “Base Deficit” (BD), and in this condition, the higher the acid, the greater the severity of the patient and the worse their prognosis^28^.

Most laboratories use BE as a standard in blood gasomestry, positive values indicating “excess of base or lack of acid”, and negative, “excess of acid or lack of base”. For example, it is positive in the presence of metabolic alkalosis and negative in acidosis, such as in states of hypoperfusion secondary to volume loss due to traumatic hemorrhagic shock^28^.

With the progressive dissemination of the BE use in clinical practice and in current literature publications, the tenth edition of Advanced Trauma Life Support, published in 2018, updated its table for the classification of hemorrhagic shock, incorporating BE as one of the parameters for such classification^2^. When the BE values in that table are applied to the present population, approximately 84% of the patients are classified as having class III and IV shock, with no class I patients at the time of ICU admission. 

According to the analysis of the ROC curve (Figure 2), the cutoff point for BE at ICU admission in this population was -10.35mmol/L, which added considerable sensitivity and specificity. Lactate had a cutoff value of 4.43mmol/L, with adequate sensitivity and specificity. These values of both parameters were higher than in the published literature. 

Also according to the ROC curve data (Figure 2), in the twenty four hour analysis, the BE cutoff point was -8.65mmol/L, also higher than the already published recommended cutoff, with a sensitivity of 54.3% and specificity of 86.5%. The lactate cutoff was 3.05mmol/L, with 65.9% and 72.2% sensitivity and specificities, respectively. Both parameters displayed very similar performances, conferring safety in clinical management with the use of BE and also with lactate. It is important to emphasize that the BE had a higher specificity than lactate both at admission and at twenty four hours, and in the case of a population of critically ill patients, having a higher specificity is extremely important, allowing the physician to be assertive and aggressive in the hemodynamic management of patients with values beyond the cutoff described in this population, as mortality and morbidity are very high if the patient remains with hypoperfusion and acidemia.

When the BE values were analyzed twenty four hours after ICU admission using a cutoff of -6mmol/L for the groups or the cutoff found in this population, the statistical significance remained valid regarding pH, bicarbonate, and lactate, reinforcing that BE can and should be used as a marker of hypoperfusion and tissue hypoxia also twenty four hours after ICU admission. Therefore, sequential follow-up at the bedside is imperative to guide hemodynamic management and volume replacement (Tables 3 and 4). 

The deleterious action of metabolic acidosis and consequent acidemia has already been well described in trauma patients, being one of the components of the lethal diamond, due to its direct interference in the coagulation cascade, greater predisposition to bradycardia and cardiac arrhythmias, in addition to the negative impact on the action of vasoactive drugs, being therefore another marker of the clinical response of patients to already instituted treatments^4^ ^-^ ^9^ ^,^ ^11^ ^,^ ^29^. 

Several studies have demonstrated the ability of BE and lactate in predicting injury severity^5^ ^,^ ^30^, predicting resuscitation end-poits^4^, playing a role in monitoring the patient’s clinical evolution, and acting as a predictor of outcome^4^. However, the speed in obtaining the BE result is an important practical advantage in the day-to-day care of this critically ill population^31^. 

There are records in the recent literature that prediction of mortality by BE is relevant at ICU admission^4^. Patients who continue with persistent hypoperfusion even after twenty four hours of adequate hemodynamic management are more likely to progress to multiple organ failure and death, which was also found in this study. 

An outcome with no statistical correlation with the BE value at ICU admission (-6mmol/L) was the need for renal replacement therapy. One limitation is that patients were not classified according to the degree of renal dysfunction, only whether or not hemodialysis was necessary. We did not study other variables, such as urea, creatinine, potassium, urine output, creatinine clearance or evolution, of these parameters during the first twenty four hours. It should be noted that urea and creatinine do not change early when it comes to acute kidney injury, especially considering a population of young patients with no previous renal dysfunction^32^.

When the BE value was evaluated after twenty four hours of ICU stay, patients who remained with BE more negative than -6mmol/L needed renal replacement therapy more than the other group, as well as when they were redivided according to the values found in this study.

Patients whose BE remained very negative, or who had BE >-10.35mmol/L at admission and maintained organic hypoperfusion within twenty four hours, required more renal replacement therapy. Hypoperfusion is also a trigger for multiple organ failure. Patients who had their perfusion improved had less dialysis, and those whise perfosion did not improve had more dialysis, with consequent higher mortality, as reported in the literature^4^ ^,^ ^33^.

The limitation of the current cohort is the retrospective nature of the data collection and the fact that it was carried out in a single center. There is also the consideration that the BE value can be altered by the consumption of alcohol^34^, a fact that can also alter the lactate value^31^, and by the infusion of a large volumes of saline solution^35^ ^,^ ^36^, variables that were not controlled. However, there is evidence that the ingestion of alcohol by the patient^26^ or the infusion of saline solution in the initial resuscitation^37^, although influencing, do not alter the validation of BE as a predictor of mortality. 

It is important to emphasize that the alterations in the values of BE and lactate due to exogenous intoxication by alcohol or drugs are relatively small and are not expressive to the point of causing diagnostic doubt in a patient in shock or occult hypoperfusion, the main diagnosis remaining tissue hypoperfusion^34^ ^,^ ^38^. 

## CONCLUSION

BE and lactate values were able to predict tissue hypoperfusion and mortality at both moments studied. BE should be monitored in the first 24 hours of ICU admission because it has good performance when compared to lactate as a predictor of mortality, being a valuable marker, with rapid results and wide availability, in addition to slightly higher specificity in both moments of the study.
